# Development, Optimization and Evaluation of 2-Methoxy-Estradiol Loaded Nanocarrier for Prostate Cancer

**DOI:** 10.3389/fphar.2021.682337

**Published:** 2021-07-16

**Authors:** Nabil A. Alhakamy, Osama A. Ahmed, Usama A. Fahmy, Hani Z. Asfour, Adel F. Alghaith, Wael A. Mahdi, Sultan Alshehri, Shadab Md

**Affiliations:** ^1^ Department of Pharmaceutics, Faculty of Pharmacy, King Abdulaziz University, Jeddah, Saudi Arabia; ^2^ Center of Excellence for Drug Research and Pharmaceutical Industries, King Abdulaziz University, Jeddah, Saudi Arabia; ^3^ Mohamed Saeed Tamer Chair for Pharmaceutical Industries, King Abdulaziz University, Jeddah, Saudi Arabia; ^4^ Department of Medical Microbiology and Parasitology, Faculty of Medicine, King Abdulaziz University, Jeddah, Saudi Arabia; ^5^ Department of Pharmaceutics, College of Pharmacy, King Saud University, Riyadh, Saudi Arabia

**Keywords:** 2-methoxyestradiol, mixed micelles, cell cycle, molecular markers, mitochondrial membrane potential

## Abstract

The therapeutic efficacy of antineoplastic agents possessing a selective target to the nucleus of the cancer cells could be enhanced through novel formulation approaches. Thus, toward the improvement of the anticancer potential of 2-methoxy estradiol (2 ME) on prostate cancer, the drug was entrapped into the hydrophobic micelles core formulated with Phospholipon 90G and d-α-tocopheryl polyethylene glycol succinate (TPGS). Optimization of the formulation was done by Box-Behnken statistical design using Statgraphics software to standardize percentages of TPGS and phospholipid to obtain the smallest particle size. The optimized formulation was found to be spherical with nanometer size of 152 ± 5.2 nm, and low PDI (0.234). The entrapment efficiency of the micelles was 88.67 ± 3.21% with >93% release of 2 ME within 24 h. There was a 16-fold increase in apoptosis and an 8-fold increase in necrosis of the PC-3 cells when incubated with 2 ME micellar delivery compared to control cells (2.8 ± 0.2%). This increased apoptosis was further correlated with increased BAX expression (11.6 ± 0.7) and decreased BCL-2 expression (0.29 ± 0.05) in 2 ME micelles treated cells when compared to the control group. Further, loss of mitochondrial membrane potential (∼50-fold) by the drug-loaded micelles and free drug compared to control cells was found to be due to the generation of ROS. Findings on cell cycle analysis revealed the significant arrest of the G2-M phase of the PC-3 cells when incubated with the optimized formulation. Simultaneously, a significantly increased number of cells in pre-G1 revealed the maximum apoptotic potential of the drug when delivered *via* micellar formulation. Finally, upregulation of caspase-9, p53, and NO, with downregulation of TNF-α, NF-κβ, and inflammatory mediators of the PC-3 cells established the superiority of the micellar approach against prostate cancer. In summary, the acquired results highlighted the potentiality of the 2 ME-micellar delivery tool for controlling the growth of prostate cancer cells for improved efficacy.

## Introduction

The second most common cancer diagnosed worldwide is prostate cancer, which is the most common solid tumor in men resulting in the second leading cancer-related deaths (Culp et al., 2020; Sabahi et al., 2020). The death cases in 2018 from 359,000 is projected to increase up to 740,000 cases in 2040, with a rise of new cases from 1,276,000 in 2018 to 2.3 million in 2040, because of increased numbers of the aging population (Culp et al., 2020). Depending on the levels of prostate-specific antigen and clinical stage of the patients, the treatment of patients with prostate cancer has opted for active surveillance, radiotherapy, or prostatectomy (Soll et al., 2020). Treatment option of prostate cancer includes androgen deprivation therapy as the mainstay management, however, the condition might progress to castration-resistant prostate cancer. The metastatic condition of castration-resistant prostate cancer is usually controlled by the use of hormonal therapies (Liu et al., 2020).

A naturally occurring estrogen metabolite, 2-methoxy-estradiol (2 ME), has shown its anti-proliferative and anti-angiogenic potential in cancer cells. This agent poorly acts on estrogen receptors, possessing no estrogenic efficacy (Harrison et al., 2011). 2 ME has shown its antitumor potential *via* induction of apoptosis through inhibition of hypoxia-inducible factor 1 and activation of p53 (Harrison et al., 2011). Other reports revealed that the binding of 2 ME to the tubulin facilitates the arrest of the mitotic cell cycle by suppressing microtubule formation (Attalla et al., 1996). A phase-II clinical report on capsule delivery for 2 ME revealed safe and adequate therapeutic potential toward decreasing PSA velocity in castration-resistant prostate cancer patients; however, it was lacking to maintain sufficient plasma concentration of 2 ME for a longer time (Sweeney et al., 2005). The advancement of the nanocrystal colloidal formulation approach of 2 ME depicted the improved pharmacokinetic profile of the agent together with antitumor potential in the animal model. However, the recommended human dose of 2 ME for the phase-II study for this nanocrystal carrier was much higher with frequent dosing (1.5 g, QID) by oral route (Sweeney et al., 2005; Tevaarwerk et al., 2009; Harrison et al., 2011).

One form of drug delivery system that has emerged in recent years is polymer-based carriers under the umbrella of nanotechnology (Gorain et al., 2018). Among different nanocarriers explored so far by different researchers, mixed micelles have gained potential interest due to the spontaneous and self-assembling property of amphiphilic copolymers into nanometric (5–100 nm) colloidal dispersions, with relatively narrow size distribution (Cagel et al., 2017; Gorain et al., 2020). The micellar structure of the carrier is spontaneously formed above the critical micellar concentration (CMC) of the polymer, thereby develop a core-shell model with the hydrophobic core to entrap the poorly water-soluble drugs with a shell constituting hydrophilic moieties (Puig-Rigall et al., 2021). The entrapped drug within the core provides the architecture for control release properties, where the hydrophilic shell stabilizes the three-dimensional spherical structure of the system (Gorain et al., 2020; Puig-Rigall et al., 2021). However, judicious selection of the polymers with biodegradable and biocompatible characteristics for the micellar delivery is of utmost importance for the safe delivery of therapeutics.

Thus, the present research has focused on developing mixed micelles for effective and safe delivery of 2 ME using Phospholipon 90G and d-α-tocopheryl polyethylene glycol succinate (TPGS). TPGS, being a derivative of natural component (vitamin E) and agreeable properties, widely incorporated in advanced drug delivery systems as a stabilizer, solubilizer, emulsifier, penetration enhancer, and protection of micelles (Guo et al., 2013; Choudhury et al., 2017). It has also shown the potential to inhibit P-glycoprotein efflux from the multidrug-resistant tumor cells with improved cellular uptake, oral bioavailability, and prolongation of circulation time (Choudhury et al., 2017). On the other hand, amphiphilicity and exceptional biocompatible characteristics make the phospholipids an appropriate component in formulation development for improved therapeutic efficacy (Singh et al., 2017). The incorporation of 2 ME within the micellar structure was optimized by design experimentation, and the optimized formulation was characterized *in vitro* for entrapment efficiency, morphology, and release characteristics. To establish the anticancer potential of the optimized formulation, cell viability, apoptosis potential, cell cycle analysis, BAX and BCL-2 estimations, and determination of molecular markers expressions were performed.

## Methodology

### Materials

2 ME (>99% purity), dialysis bag (molecular weight cut-off 12,000), 3- (4,5-dimethyl-thiazol-2-yl)-2,5-diphenyl-tetrazolium bromide (MTT) and TPGS were procured from Sigma-Aldrich, United States. Phospholipon 90G, American Lecithin Company, CT, United States HPLC grade acetonitrile and methanol were procured from Sigma, United States. Trizol was purchased from Invitrogen, PA, United Kingdom. The rest of the chemicals used in this project were of analytical grade.

### Experimental Design for Optimization of Formulation Using Statgraphics

Using of Box-Behnken statistical design (Statgraphics Technologies, Inc. VA, United States), an RSM tool, has been introduced in the optimization of pharmaceutical formulations using experimental trials. In the present experiment, three factors at their three levels model was used to optimize the 2 ME mixed micelles. Based on the literature, maximum and minimum levels of the three independent variables, such as phospholipid (Phospholipon 90G), TPGS, and stirring time were fed in the software for identifying the optimized mixed micelles with suitable particle size using the Quality by Design technique (Kumbhar et al., 2021). Based on the information fed in the software, it suggested 15 batches of mixed micelles formulations with a different combination of independent variables at low (−1), medium (0), and high (+1) levels. The batches of formulation were developed according to the method described in *Method of Preparation of Mixed Micelles* by varying the independent variables mentioned in Table 1. Those fifteen 15) batches of formulations suggested were with varied concentrations of phospholipid (1–3 mg/ml), TPGS (10–30 mg/ml), and stirring speed (1–5 min). Following the development of the formulations, the obtained dependent variables, i.e., particle sizes for the different batches of formulation were determined following the method described in *Determination of Particle Size and Polydispersity*. Upon determining the particle size of the mixed micelles, the data (Table 1) were included in the software to achieve the composition of the optimized formulation with a suitable size. Further, statistical analysis was performed in the software using analysis of variance (ANOVA) to evaluate the significance of the studied variables. Additionally, the interaction between the independent variables was represented using interaction plot, Pareto plot, and contour plot (Md et al., 2020).

**TABLE 1:** Experimental runs in Box–Behnken statistical design: The independent variables and experimental dependent variable (particle size).

Run	Values of independent variables			Dependent variable (Y)	
	A	B	C	Particle size (observed)	Particle size (fitted)
1	3	10	3	453.0	438.375
2	3	20	1	411.0	404.5
3	2	20	3	254.0	256.333
4	3	20	5	382.0	380.25
5	2	30	1	241.0	224.625
6	1	30	3	176.0	190.625
7	2	10	5	199.0	215.375
8	1	20	5	141.0	147.5
9	1	10	3	110.0	87.125
10	2	30	5	228.0	206.875
11	1	20	1	241.0	242.75
12	2	10	1	296.0	317.125
13	2	20	3	264.0	256.333
14	3	30	3	211.0	233.875
15	2	20	3	251.0	256.333

Independent variable	Levels		
	Low (−1)	Medium (0)	High (1)
Phospholipid (A) (mg/ml)	1	2	3
TPGS (B) (mg/ml)	10	20	30
Stirring time (C) (min)	1	3	5
Dependent variables: Y = particle size			

### Method of Preparation of Mixed Micelles

The preparation of mixed micelles for 2 ME was performed following the report by Ahmed and team (Ahmed et al., 2019), where the emulsion method was adopted in the preparation of the micellar preparation. In brief, Phospholipon 90G, TPGS, and 2 ME were dissolved in ethyl alcohol using a magnetic stirrer for 5 min. Thereafter, purified water was added to the prepared solution. Finally, the micellar dispersion was developed by evaporating the organic solvent (ethyl alcohol) using a round bottom flask in a rotary evaporator. Finally, the prepared dispersion of the components was centrifuged at 30,000 rpm at 4°C for 30 min. Later, lyophilization was done of the residue using a freeze dryer (Martin Christ Gefriertrocknungsanlagen GmbH, Osterode am Harz, Germany) obtained after the centrifugation method. After 2 days of the drying process, the samples were stored for further characterization.

### Evaluation of 2 Methoxy Estradiol Loaded d-α-Tocopheryl Polyethylene Glycol Succinate-Phospholipid Micelles

#### Determination of Particle Size and Polydispersity

Measurement of particle size of the developed 2 ME-loaded TPGS-phospholipid micelles was accomplished using a laser diffraction particle size analyzer (Zetasizer Nano ZSP, Malvern Panalytical, United Kingdom), where the size and polydispersity of the micelles were determined at 25°C following an equilibrating period of 2 min.

### Micellar Microscopy Investigation

The developed optimized 2 ME-loaded micelles was investigated using a transmission electron microscope TEM (JEOL JEM-HR-2100, JEOL, Ltd. Tokyo, Japan). In due course, a drop of the diluted sample in purified water was placed on the copper grid and then stained with 2% uranyl acid. The observation of the grid was made after drying at room temperature (Alfaifi et al., 2020).

### Entrapment Efficiency

The entrapment efficiency of the developed optimized 2 ME-loaded mixed micelles was determined dissolving the formulated micelles in methanol and the content of 2 ME was analyzed by RP-HPLC method using C18 column (4.6 mm × 150 mm, 5 µm). The separation of 2 ME was achieved using the mobile phase consisting of acetonitrile and methanol at a ratio 55:45 (*v/v*) at a wavelength of 285 nm (Du et al., 2010). Finally, the percentage of entrapment efficiency was determined using the following Eq. 1:

$$Entrapment\;efficiency\left(\%\right)=\frac{Weight\;of\;2ME\;in\;the\;optimized\;formulation}{Weight\;of\;2ME\;in\;the\;dispersion\;}\times 100$$ (1)

#### *In vitro* Release

The release pattern of 2 ME from the optimized micellar delivery system was determined by the dialysis bag method (Ponta et al., 2015), where the sample containing 5 g of 2 ME was loaded into the activated dialysis bag (12 kDa cut-off) and closed both the ends hermetically. Thereafter the release of 2 ME from the setup was determined in paddle-type dissolution apparatus, where phosphate buffer saline (pH 7.4) was used as releasing media. The paddle was set at 100 rpm and the temperature of the system was maintained at 37 ± 0.5°C. A volume of 10 ml sample was withdrawn at each time point at 0, 2, 4, 6, 8, 10, 12, 18, and 24 h. Analysis of 2 ME within the withdrawn sample was performed using the HPLC method (Du et al., 2010).

### Cell-Based Evaluation of the Optimized Formulation

#### Cytotoxicity Assay

The cytotoxicity analysis of the free 2 ME blank micelles and the formulated 2 ME-loaded mixed micelles in PC-3 cell line was evaluated following the MTT assay (Akrawi et al., 2020). Maintaining different drug concentrations in the 96-well plate containing 5×10^3^ cells onto each well were incubated for 24 h at 37°C. The wells were washed with phosphate buffer saline (pH 7.4) after the incubation period. Thereafter, 0.5% (*w/v*) MTT solution was added to the wells and incubated for another 4 h at 37°C and 5% CO_2_ until a purple precipitate was visible prior to analysis. Then, the cells were washed again to remove the access MTT solution from the cells and 150 µL of DMSO was added to each well to dissolve the formed formazan crystals. Finally, the viability of the cells was determined by the results on optical density of the wells containing purple colored liquid at 490 nm when examined in a microplate reader.

### Apoptotic Assay

We have adopted the double staining technique to establish the apoptotic potential of the blank micelles, free 2 ME, and the formulated 2 ME-loaded mixed micelles in PC-3 cell line. In brief, the PC-3 cells were exposed to the samples in a 12-well plate, where the initial density of the seeded cells was maintained at 10^5^ cells per well (Zhang et al., 2020). IC50 concentration of 2 ME was maintained in the wells, which were incubated at 37°C for 24 h. Thereafter, the cells were washed with phosphate buffer in order to remove the micelles and free drug from the wells, and the apoptotic potential of the agents was measured using the conjugate of Annexin V–fluorescein isothiocyanate (FITC) and propidium iodide following the instruction provided with the apoptotic detection kit (BD Bioscience, United States). Finally, the flow cytometer (FACS Calibur, BD Bioscience) was used to analyze the cells, and the obtained data were analyzed using the Multicycle software (Phoenix Flow Systems, San Diego, CA).

#### Cell Cycle Analysis

Analysis of cell cycle was done by the treatment of blank micelles, free 2 ME and the formulated 2 ME-loaded mixed micelles with 1 × 10^6^ numbers of PC-3 cells per well. Following treatment of 24 h, the treated cells were trypsinized for detaching the cells from the respective wells. The cells were collected and washed with phosphate buffer and fixed again in a separate well plate. Thereafter, the cells were evaluated for arrest in the cell cycle using the TESTTM PLUS DNA reagent kit (BD Biosciences, San Jose, CA) and following the instructions from the manufacturer. Finally, the testing of cell cycle distribution was assembled using a flow cytometer (FACS Calibur, BD Bioscience) and analyzed using the Multicycle software (Phoenix Flow Systems, San Diego, CA).

#### BAX and BCL-2 Estimation Using Real-Time PCR (Rt-PCR)

The RT-PCR technique was incorporated to evaluate the BAX and BCL-2, where the PC-3 cells were incubated with the blank micelles, 2 ME-loaded micelles, and free drug (2 ME) for 48 h. Thereafter, the cells were lyzed using trizol reagent. Thereafter, the RNA was extracted from the cells following the standard protocol available with the kit instruction (Qiagen, Germany). Subsequently, extracted RNA was used to produce cDNA using the instructions of cDNA kit (Qiagen, Germany). Finally, the RT-PCR of the samples was performed using the commercially available kit using the following conditions: 95°C for a period of 10 min followed by 40 cycles consisting of 95°C for 15 s and 60°C for 60 s. The primers used in this experiment were GCT​GGA​CAT​TGG​ACT​TCC​TC and ACC​ACT​GTG​ACC​TGC​TCC​A (forward and reverse sequences 5ʹ to 3ʹ, respectively) for Bax and GGA​TGC​CTT​TGT​GGA​ACT​GT and TCA​CTT​GTG​GCC​CAG​ATA​GG (forward and reverse sequences 5ʹ to 3ʹ, respectively) for Bcl-2 (Khorsandi et al., 2020).

#### Determination of Mitochondrial Membrane Potential

Effect of the blank micelles, free 2 ME, and the formulated 2 ME-loaded mixed micelles on mitochondrial membrane potential was determined on PC-3 cells following Zhang et al. (Zhang et al., 2011). In due course, the treated cells were washed with phosphate buffer saline (pH 7.4) to remove the remaining samples. Thereafter, the cells were incubated with rhodamine-123 (10 μg/ml) for 10 min. The treated cells were washed again with phosphate buffer thrice and the cells were placed into the six-well plate. The cells were allowed to grow for 60 min. After the incubation period, the fluorescence intensity of the cells was measured following exposure of excitation and emission wavelengths at 488 and 530 nm, respectively using a fluorophotometer.

#### Molecular Marker Estimation Using ELISA Method

In order to determine the molecular markers within the treated cells, PC-3 cells of different groups were collected following treatment, trypsinization and centrifugation at 2,500 rpm for 5 min. Thereafter, the cells were washed with phosphate buffer saline (pH 7.4) twice and then lyzed using ice-cold radio immune-precipitation buffer following incorporation of protease inhibitor (Balachandran et al., 2014). The levels of caspase-9, p53, TNF-α, NF-κβ, COX-2, IL-6, and NO within the cells were measured following the manufacturer’s instructions in the commercially available kits (Invitrogen, United States) (Mohamed et al., 2020).

### Statistical Analysis

Using the graphpad prism software, the obtained data analysis was completed. The experiments were carried out in triplicate and the results are presented as mean ± standard deviation. The statistical data analysis was carried out using the one-way analysis of the variance (ANOVA) test followed by Tukey post hoc multiple comparison test, where the *p*-value less than 0.05 was regarded as significant.

## Results and Discussion

### Optimization of Mixed Micelles Formulation

In the fabrication of the optimized formulation in the pharmaceutical field, the design of experiments and statistical analysis are widely incorporated. In the present experiment, the size of the developed mixed micelles was set as the dependent variable whereas the composition of TPGS and phospholipid and the stirring time were set as the independent variables. From the obtained results it could be seen that quadratic processing of independent variables has a significant effect on the dependent variables. The statistical outcome of the interaction of phospholipid (A) and TPGS (B) content and stirring time (C) on the particle size of the 2 ME-loaded micelles is presented in Table 2, where the statistical significance of the interaction of A, B, and C on particle size is denoted by the *p* values of less than 0.05.

**TABLE 2:** Analysis of variance data for particle size.

Source	Sum of Squares	F-Ratio	p-Value
A: Phospholipid	77,815.1	126.06	0.0001
B: TPGS	5,100.5	8.26	0.0348
C: Stirring time	7,140.13	11.57	0.0192
A^2^	1,061.85	1.72	0.2467
AB	23,716.0	38.42	0.0016
AC	1,260.25	2.04	0.2124
B^2^	4,730.01	7.66	0.0395
BC	1764.0	2.86	0.1517
C^2^	1,545.39	2.50	0.1744

The *p*-value in the table (Table 2) indicated that model terms A, B, C, AB, and BB are significant as the *p-*value of the model terms is < 0.05. On the other hand, the observed and fitted values for the particle sizes of 2 ME-loaded mixed micelles are in close agreement, as represented in Table 1. Therefore, the difference between the fitted value (as predicted by the software) and the actual measured value of the developed formulation by the nano-sizer instrument is very low which indicated the suitability of the data with the design. Further, the percent error represented the percent difference in observed and fitted values are very low.

**FIGURE 1:**
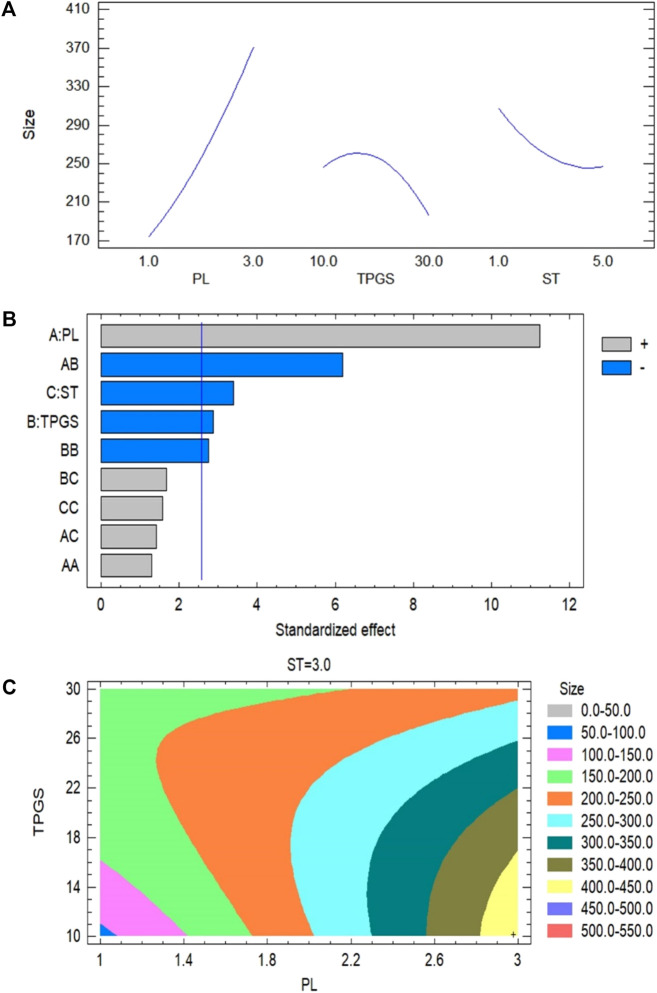
**(A)** Main plot effect on the interaction of phospholipid, TPGS contents and stirring time on the particle size of 2 ME-loaded micelles formulation. **(B)** Pareto chart on the interaction of phospholipid, TPGS content and stirring time on the particle size of 2 ME-loaded micelles formulation. **(C)** Contour plot on the interaction of phospholipid, TPGS content and stirring time on the particle size of 2 ME-loaded micelles formulation.

A polynomial equation presented the effect of the independent variables (A, B, and C) on the particle size of the 2 ME-loaded micelles (Eq. 2). A positive coefficient with a value of +158.167 is observed with the model term A, which is the highest coefficient when compared to the other model terms. It indicates that there will be an increase in particle size with the increasing content of phospholipid. Alternatively, the negative coefficient of model term C with the value of −84.375 indicated that the increase in stirring time will lead to a decrease in particle size of the developed 2 ME-loaded micelles. A similar effect of phospholipid, and stirring time on particle size is reflected in the main effect plot (Figure 1A). In the figure, the sharp positive slope of the curve associated with model term A denoted that the model term A has the maximum effect. Further, the effect is proportional to the particle size. The negative slope of the model term C in the main effect plot effect (Figure 1A) is at per the coefficient represented in Eq. 2. The obtained results from Eq. 2 and main effect plot on the effect of model terms A and C on the particle size of a 2 ME-loaded mixed micelles are further confirmed in the Pareto plot, where the maximum effect of phospholipid with the longest bar diagram of the model term A with a positive response is clearly presented, i.e., increasing A will lead to increasing particle size. In contrast, an inverse effect is presented with model term C. Alternatively, the positive coefficient of model term B is not as per the main plot effect and Pareto chart (Figures 1A,B). In the main plot effect, the positive slope followed by a negative slope, which indicated that an initial increase in TPGS concentration will result in an increase in the particle size of the formulation; however, further increase in TPGS concentration will lead to a decrease in particle size. The initial positive slope is reflected in the coefficient value (+24.0417) of model term B in Eq. 2. Furthermore, the negative slope in the main plot effect is at per the Pareto chart where the negative effect of TPGS is represented on particle size. Additionally, the greater coefficient value of the model term C compared to the model term B in Eq. 2 is in accordance with a longer bar diagram associated with model term C than the model term B in the Pareto chart (Figure 1B).

From the contour plot on particle size it could be said that the maximum effect is presented for the model term A, which has further been established by the maximum color changes through the model term A-axis from blue to yellow. This color change indicated that the change in particle size approximately from 50 to 450 nm with changes of model term A in different runs (Figure 1C). Alternatively, color changes through the TPGS axis are only limited to blue to light green, which indicated the changes of particle size with changes of TPGS concentration approximately ranged from 50 to 200 nm and with the model term C, changes of particle size were ranged from 150 to 250 nm. Further, our findings are in agreement with the literature where it is evident that increasing phospholipid leads to increasing particle size of micelles whereas increasing TPGS concentration leads to decreasing particle size of the fabricated micelles (Chen et al., 2016). Further, the decrease in particle size with the increase in TPGS concentration might be due to the surfactant effect of the agent (Jin et al., 2018).

$$\text{Y}\left(\text{Size}\right)=\;-66\text{.}6563\;+\;158\text{.}167\text{A }+\;24\text{.}0417\text{B }-\;84\text{.}375\text{C }+\;16{\text{.}9583\text{A}}^{2}\;-\;7\text{.}7\text{AB }+\;8\text{.}875\text{AC }-\text{0}{\text{.}357917\text{B}}^{2}\;+\;1\text{.}05\text{BC }+\;5{\text{.}11458\text{C}}^{2}\left(2\right)$$

Based on the software analysis, we had set the minimize size as the goal to obtain the optimized formula for the development of the optimized micelles. From the runs in the software, based on the high to low range of phospholipid concentration (1–3 mg/ml), TPGS concentration (10–30 mg/ml), and stirring time (1–5 min), it has given the optimized formula with 1.0226 mg/ml of phospholipid, 10.0% TPGS and 5 min of stirring time for the optimized formulation. Using the optimized formula, the micellar formulation was fabricated and characterized for the following parameters.

### Evaluation of 2 Methoxy Estradiol Loaded d-α-Tocopheryl Polyethylene Glycol Succinate-Phospholipid Micelles

#### Determination of Particle Size, Polydispersity, and Morphology of the Optimized Formulation

The particle size of the formulated and optimized 2 ME-loaded mixed micelles, as determined by the light scattering method, was found to be 152 ± 5.2 nm, whereas the PDI of the formulation was found to be 0.234. The PDI of the formulation is a measure of the size homogeneity of a batch of the sample, where low PDI reflects a low level of aggregation or agglomeration of the samples (Mudalige et al., 2018). Thus, the obtained low PDI of the formulated micelles indicated homogenous distribution of the particles. Further, according to the reported literature, the nanometric size (<200 nm) of the carriers supports longer circulation within the body facilitating passive targeting *via* enhanced permeation and retention effect to the vascularized and fenestrated cancerous environment (Choudhury et al., 2019b).

**FIGURE 2:**
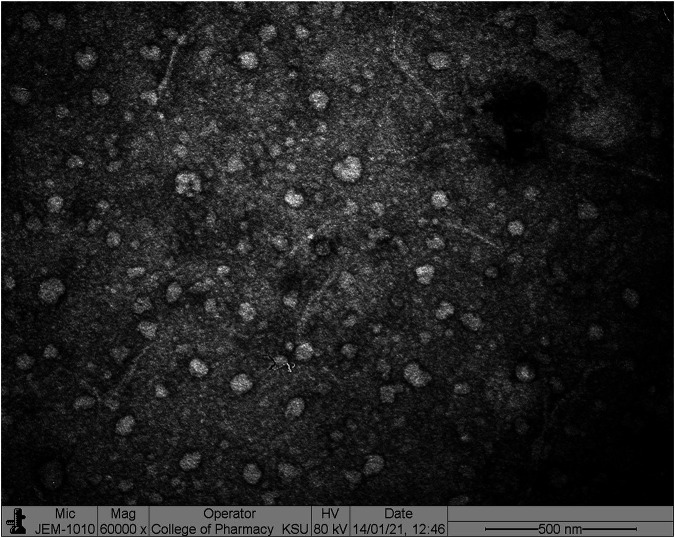
Optimized 2 ME-loaded mixed micelles investigated by TEM.

On the other hand, the TEM image of the optimized formulation, as displayed in Figure 2, indicated the spherical morphology of the particles. The size of the particles slightly less than 150 nm as obtained in TEM analysis was found to be comparable to the size obtained in the light scattering method. The homogeneity of the particle size of the formulated micelles was also confirmed from Figure 2.

#### Entrapment Efficiency

Entrapment of the drug within the formulated mixed micellar structure was found to be 87.23 ± 3.54%, which indicates that, the entrapment of 2 ME within the mixed micelles is high enough due to the entrapment of the drug within the hydrophobic core of the micellar structure. This might be correlated to the solubility enhancement property of TPGS because of its amphiphilic characteristics due to the polar terminal group with a hydrophobic long carbon chain (Choudhury et al., 2017; Gorain et al., 2018). Our result of increased entrapment efficiency by the presence of TPGS within the micellar structure is in agreement with the existing literature, where 88.67 ± 3.21% entrapment of ellagic acid within the TPGS micelles was reported by the authors (Alfaifi et al., 2020).

#### *In vitro* Release Study

**FIGURE 3:**
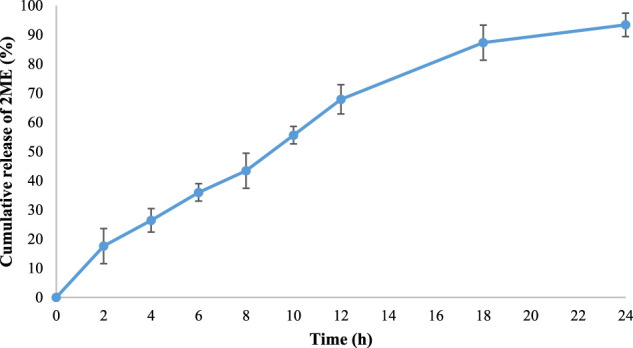
The cumulative release pattern of 2 ME from the optimized 2 ME-loaded mixed micelles in phosphate buffer (pH 7.4). Values are expressed as mean ± SD (*n* = 3).

The release pattern of 2 ME from the formulated mixed micelles formulation was portrayed in Figure 3. From the figure, it could be observed that the diffusion rate of 2 ME from the micellar delivery was almost constant, providing controlled release characteristics within the physiological pH. A total of 93.4 ± 3.63% release was observed within the time frame of 24 h. In contrast, the release of 2 ME from the crude powder was reported to release approximately 20% in phosphate buffer (pH 7.4) as evidenced in the literature (He et al., 2019). Therefore, the increased release characteristics of 2 ME from the developed and optimized micellar delivery tool could be inferred that the developed nanocarrier aids in improving the pattern of release of poorly water-soluble drugs (Lu and Park, 2013; Choudhury et al., 2014).

### Cytotoxicity Assay

**FIGURE 4:**
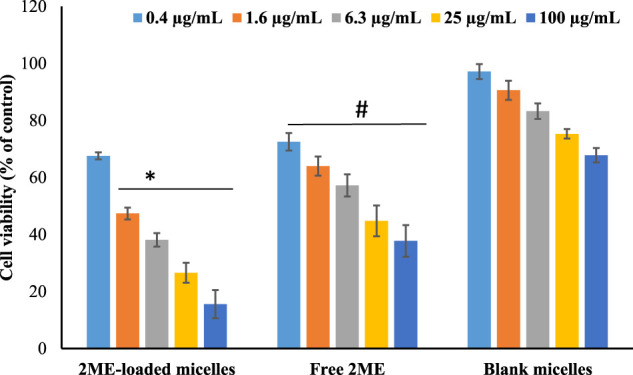
Cell viability of the cells treated with 2 ME-loaded mixed micelles, free drug (2 ME) and blank micelles were presented. Control group is 100% cell viability. Values are expressed as mean ± SD (*n* = 3). * represents significant difference between 2 ME-loaded mixed micelles (*p* < 0.05) vs. blank micelles and free 2 ME [except for 0.4 μg/ml for (*p* > 0.05). # represents significant difference between free 2 ME vs. blank micelles (*p* < 0.05)].

The finding of MTT assay on PC-3 cell line following treatment of blank micelles (no drug/empty micelles), free drug (2 ME), and drug-loaded mixed micelles is presented in Figure 4. It is clearly depicted that there is a dose-dependent decrease in the cell viability for all the treatments; however, the maximum effect of the drug-loaded mixed micelles is clearly evident. It is also reflected by the significant decrease (*p* < 0.05) of IC50 value of drug-loaded micellar delivery (1.98 ± 0.18 μg/ml) when compared to the free drug (13.09 ± 5.95 μg/ml), calculated by nonlinear regression method. Cytotoxicity of blank formulation might be explained by the presence of TPGS, as literature is evident for the anticancer potential of TPGS against prostate and lung cancer (Neophytou and Constantinou, 2015). Therefore, the improved cytotoxic potential of the micellar approach of 2 ME could be explained by the fact of the improved release of 2 ME from the micelles, improved penetration of the drug through the cell membrane because of the nanometric size range (Choudhury et al., 2019a; Faramarzi et al., 2019) together with the cytotoxic potential of TPGS against prostate cancer cells (Neophytou and Constantinou, 2015). Enhancement of cell penetration and cytotoxic potential of 2 ME is evident in literature when delivered *via* nanoformulation (Wang et al., 2011).

### Apoptotic Assay

**FIGURE 5:**
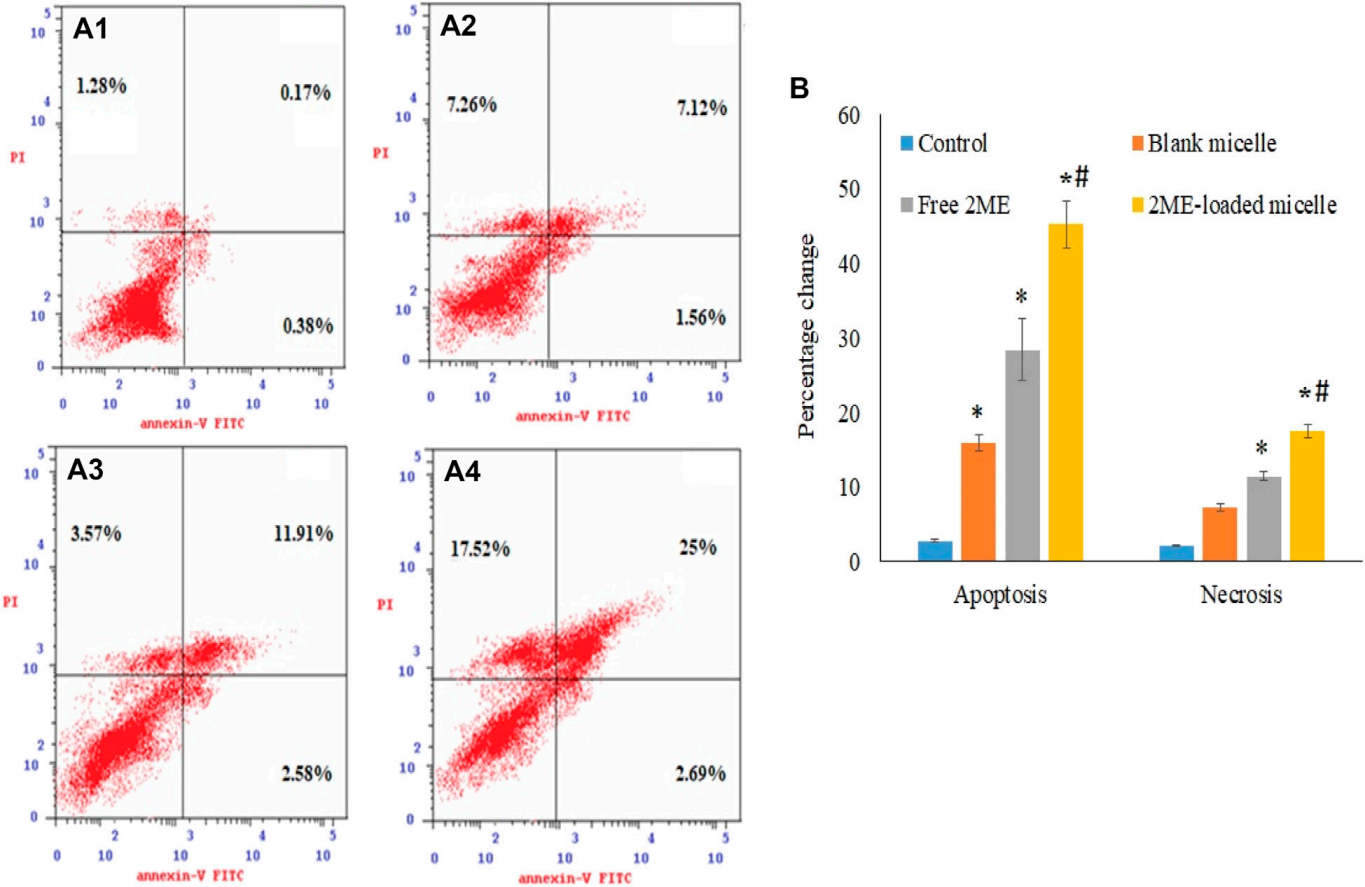
Apoptotic and necrotic assessment of blank-micelles, free 2 ME, and 2 ME-loaded micelles in PC-3 cell line. The cells were exposed to the samples for 24 h and stained with Annexin-V/FITC and propidium iodide, control **(A) (i)**, blank micelles **(ii)**, free 2 ME **(iii)**, and 2 ME-loaded micelles **(iv)**. **(B)** Representation of PC-3 cell death following apoptotic and necrotic assay by cytometric analysis after annexin V staining. Values are expressed as mean ± SD (*n* = 3). * represents significant difference from control group (*p* < 0.05). # represents significant difference between 2 ME-loaded micelles vs other groups.

The apoptosis process is a genetically triggered, programmed cell death process to control and maintain the healthy condition of the body, whereas, premature death of the cells is represented by necrosis. Herein, the apoptotic potential of the blank micelles, drug-loaded micelles, and the free drug is presented in Figures 5Ai–iv,B. Significant (*p* < 0.05) increase in apoptosis in the blank micelles treatment group might be explained by the apoptotic potential of TPGS. According to the literature, this TPGS in the blank micelles possesses the potential to increase ROS load within the cancer cells, facilitating apoptosis of the cancer cells (Singh et al., 2019). Incubation of prostate cancer cells with all tested samples for a period of 24 h showed a significant increase (*p* < 0.05) in apoptotic potential when compared to the control cells (2.8 ± 0.2%). However, when the effect of micellar delivery of 2 ME (45.2 ± 3.2%) was compared to free 2 ME (28.4 ± 4.0%), it was found to be a significant increase in apoptosis (*p* < 0.05) when the cells were treated using micellar delivery approach. This could be correlated to the increased release of drug together with increased penetration of the nanocarrier to the cancer cells (Choudhury et al., 2019a; Faramarzi et al., 2019).

Similarly, micellar deliveries and the free drug treatment to the PC-3 cells revealed a significant increase in necrosis (*p* < 0.05) when compared to the necrosis in control cells (2.14 ± 0.12%), which correspond to the apoptosis efficacy of the treatments on the cancer cells (Figure 5B).

### Cell Cycle Analysis

**FIGURE 6:**
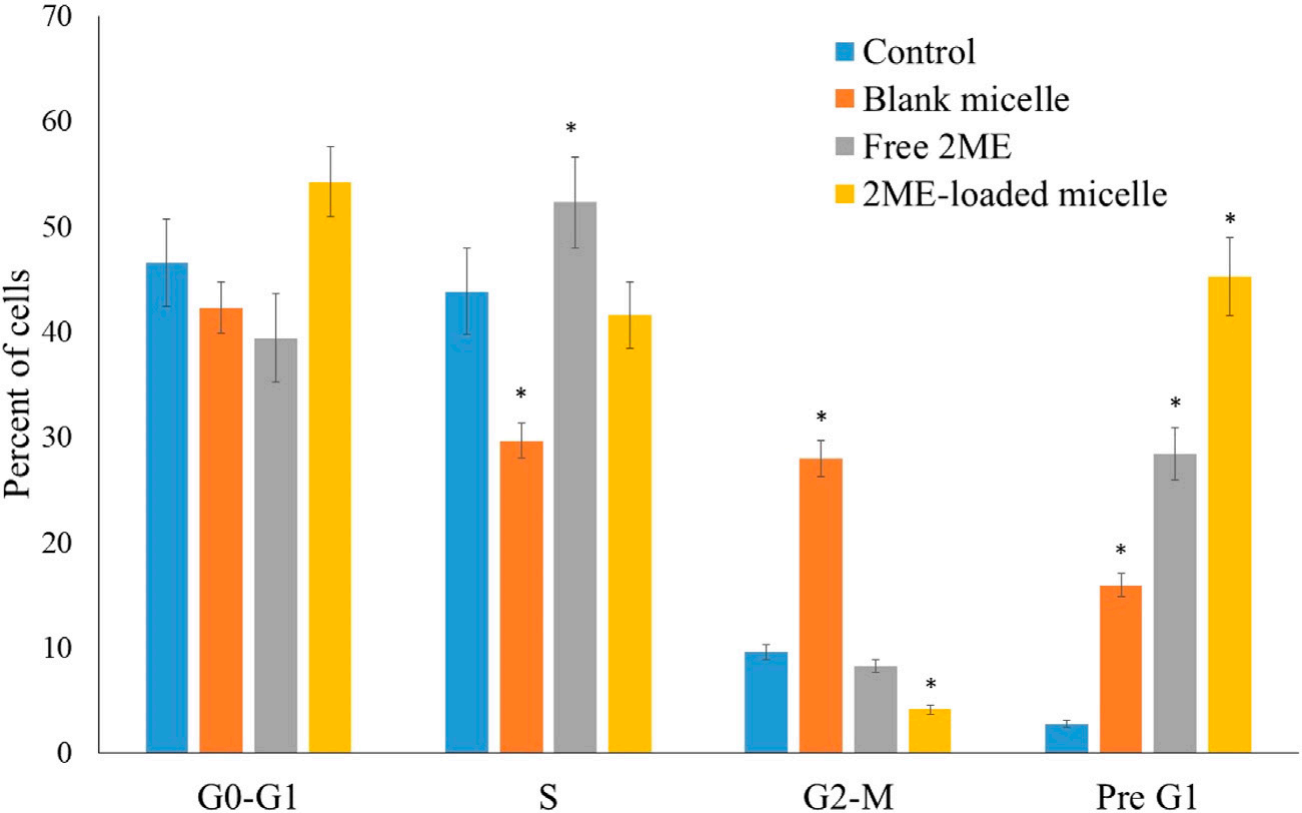
Flow cytometric analysis of blank micelles, free-2ME, and 2 ME-loaded micelles on the cell cycle distribution of PC-3 cells. * represents significant difference from control group (*p* < 0.05). Values are expressed as mean ± SD (*n* = 3).

The effect on the growth of PC-3 cells by the blank micelles, free 2 ME, and 2 ME-loaded micelles was determined by DNA content flow cytometric assay. The findings are presented in Figure 6. The micellar formulation of 2 ME was found to induce arrest of the G2-M phase of the cell cycle. The reduction of G2-M from 13.86% because of free drug treatment was increased by 56.93, which might be correlated with the increased apoptotic potential of the optimized micellar formulation. Our results on arresting G2-M phase by the action of 2 ME are comparable to the reported literature (Lee et al., 2008); however, an increased level of arrest could be justified by the formulation approach. Further, the increase of cells in the G2-M phase when treated with blank-micelles could be correlated to the decreased cell count in the S-phase. Thus, the apoptotic potential of the blank micelles could be correlated to the arrest of the cell cycle at the S-phase (Shangguan et al., 2014). On the other hand, there is a significant increase of cells in the pre-G1 phase (*p* < 0.05) when compared to the cells in the control group, or cells in the blank micelles. The increased numbers of cancerous cells in the pre-G1 phase could also be related to the existing literature (Lee et al., 2008). The effect on the G0-G1 and S phases of the cell cycle by the free drug and drug-loaded micelles is not significant. A characteristic sign of apoptotic potential is an increased percentage of cells in the pre-G1 phase of the cell cycle (Anwar et al., 2019). In addition, the induction of apoptosis and moderation of different cell cycle stages may be associated with increased cytotoxicity of the micellar delivery of 2-ME, which might modulate expressions of the cellular component during cell signaling pathways of apoptosis and cell cycle arrest. This could be correlated to the findings of the next experiment.

### BAX and BCL-2 Estimation

**FIGURE 7:**
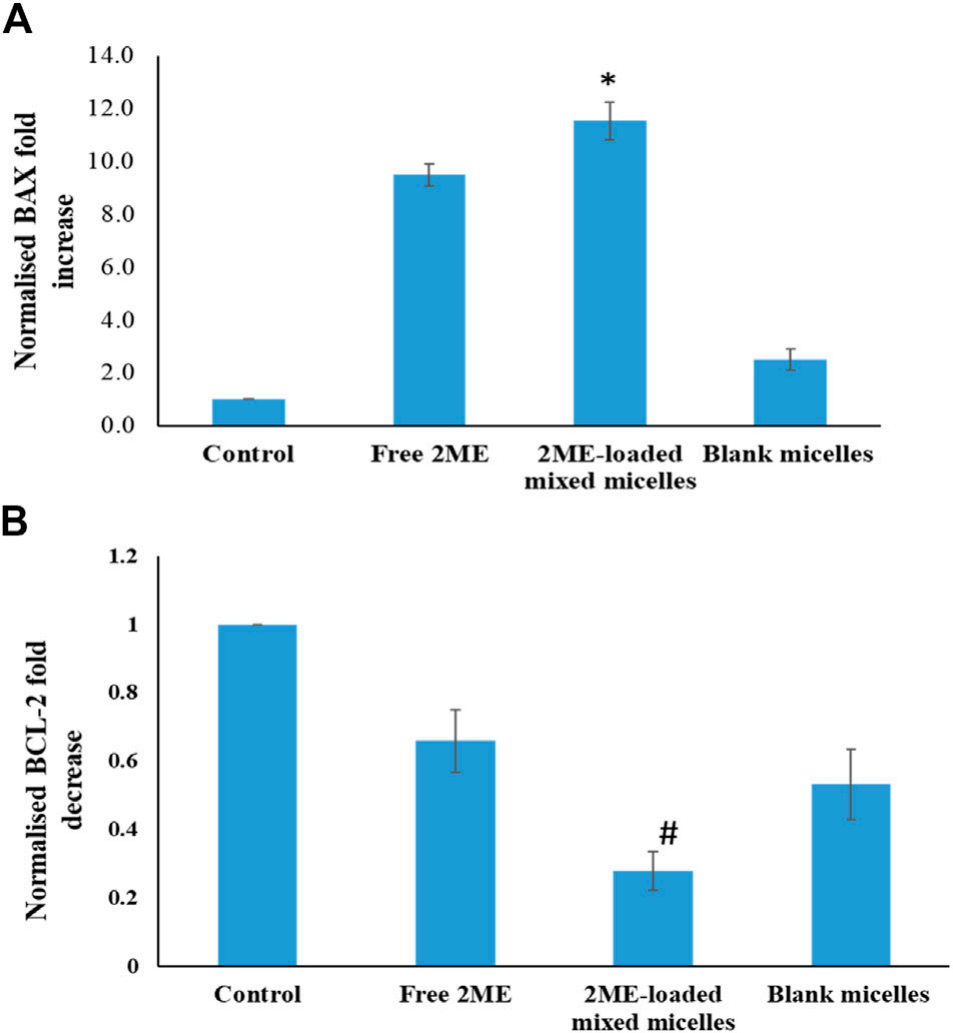
A. BAX and B. BCL-2 expression levels of the blank micelles, free-2ME, and 2 ME-loaded micelles exposed PC-3 cells and vehicle control cells after 24 h. BAX and BCL-2 expression levels were normalized with regard to control cells. * represents significant difference between 2 ME-loaded micelles vs other formulations group (*p* < 0.05). Values are expressed as mean ± SD (*n* = 3).

Induction of BAX protein production leads to the increased apoptosis, whereas BCL-2 is known to be an anti-apoptotic protein (Yang et al., 2015). RT-PCR based estimation results on expression of BAX and BCL-2 are presented in Figure 7, where the significant increase of BAX protein expression (*p* < 0.05) in the PC-3 cells could be correlated with the increased apoptosis in the 2 ME treated cells. Further, increased apoptosis in 2 ME-loaded micelles-treated cells might be correlated to the highest expression level of BAX in the PC-3 cells treated with 2 ME-loaded micelles. In contrast, there was a significant decrease (*p* < 0.05) in BCL-2 protein expression in the PC-3 cells when treated with the 2 ME-loaded micelles as compared to control. Thus, a significant decrease in BCL-2 levels reflected by the increased apoptosis levels in free drug and drug-loaded micelles treated cells. Reports available in the literature support our findings on the expression of BAX and BCL-2 protein in cancer cells by the action of 2 ME (Joubert et al., 2005b; 2005a). Alternatively, treatment of blank micelles also showed significant (*p* < 0.05) moderation of BAX and BCL-2 protein expression. These results could be correlated to the increased level of apoptosis and necrosis within the blank micelles treated cells. An increase in apoptotic potential with a simultaneous change in BAX and BCL-2 level had also been reported for TPGS-based carrier (Li et al., 2016).

### Mitochondrial Membrane Potential

**FIGURE 8:**
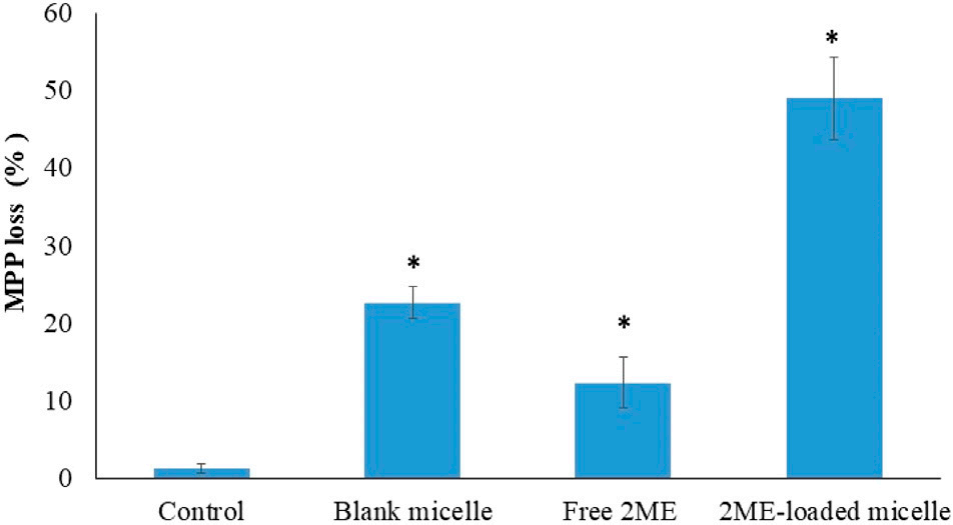
Loss of mitochondrial membrane potential (MMP) of the PC-3 cells following incubation with blank micelles, free-2 ME and 2 ME-loaded micelles and vehicle control cells. * represents significant difference from control group (*p* < 0.05). Values are expressed as mean ± SD (*n* = 3).

The role of 2 ME on experimental cells had revealed a dose-dependent increase in reactive oxygen species (ROS), which, in turn, resulted in mitochondrial membrane potential (Zhang et al., 2011). Our result on mitochondrial membrane potential following treatment of PC-3 cells with blank micelles, free 2 ME, and 2 ME-loaded micelles is presented in Figure 8. From the figure, it could be inferred that the free drug and the micellar approach of 2 ME showed a significant increase significant (*p* < 0.05) in mitochondrial membrane potential when compared to the results of control cells, which might be because of increased generation of ROS. Our results of increasing mitochondrial membrane potential together with increased apoptosis of the experimental cells are in agreement with the existing literature (Gao et al., 2006; Zhang et al., 2011). Thus, the increased ROS within the cancer cells might have increased the oxidative stress of the cells, leading to increased loss of mitochondrial membrane potential. Similarly, TPGS had also been reported to generate ROS within the treated cells (Singh et al., 2019), which ultimately induce apoptosis of the cells. Our results on the significant increase in mitochondrial membrane potential by the treatment of blank micelles might be because of increasing ROS by the action of TPGS in the formulation.

### Molecular Marker Estimation Using ELISA Method

**FIGURE 9:**
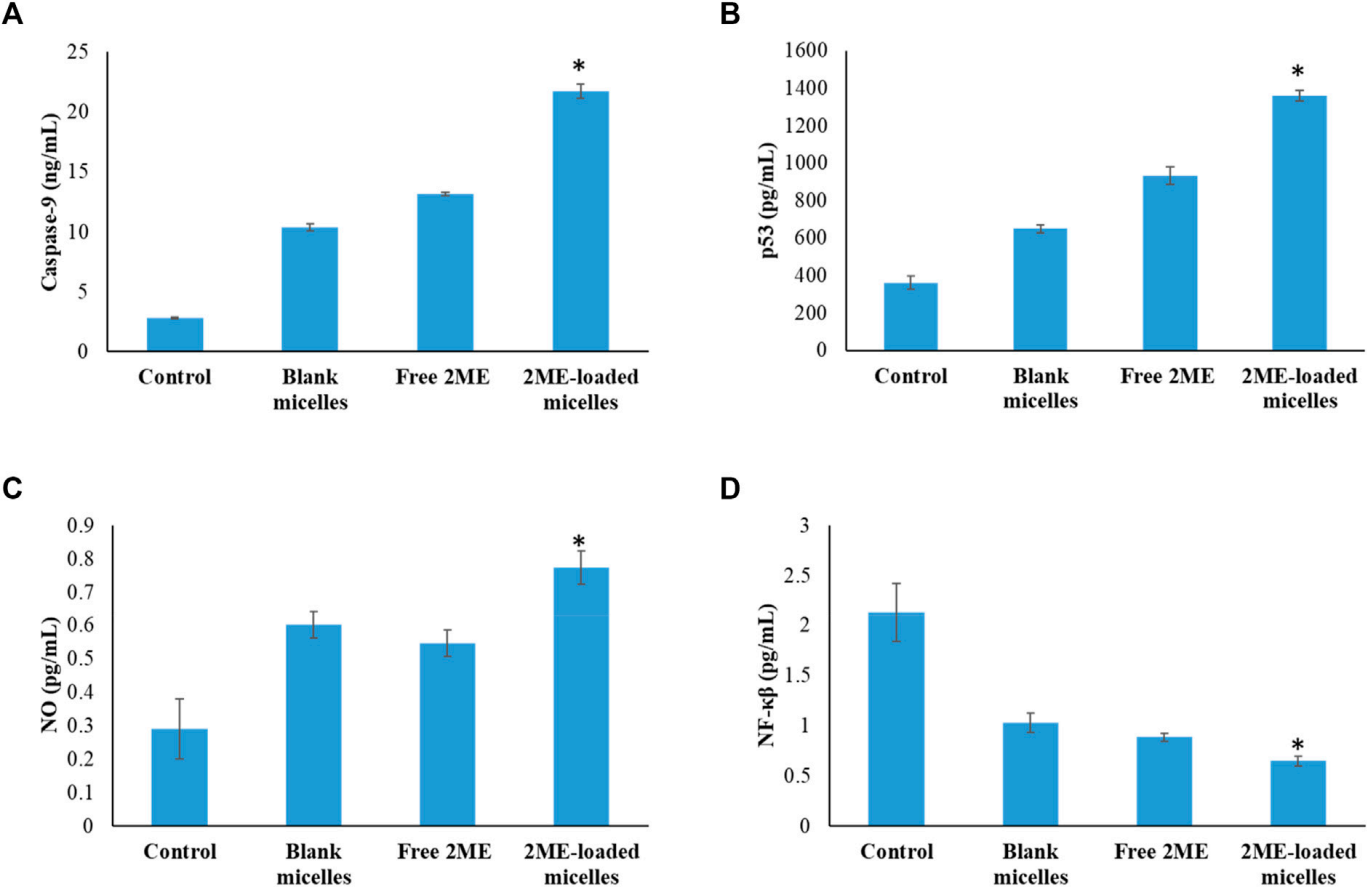
Change in the expression levels of caspase-9 **(A)**, p53 **(B)**, NO **(C)**, and NF-κβ **(D)** on the PC-3 cells following incubation with blank micelles, free-2 ME, and 2 ME-loaded micelles and vehicle control cells. * represents significant difference between 2 ME-loaded micelles vs other formulations group (*p* < 0.05). Values are expressed as mean ± SD (*n* = 3).

**FIGURE 10:**
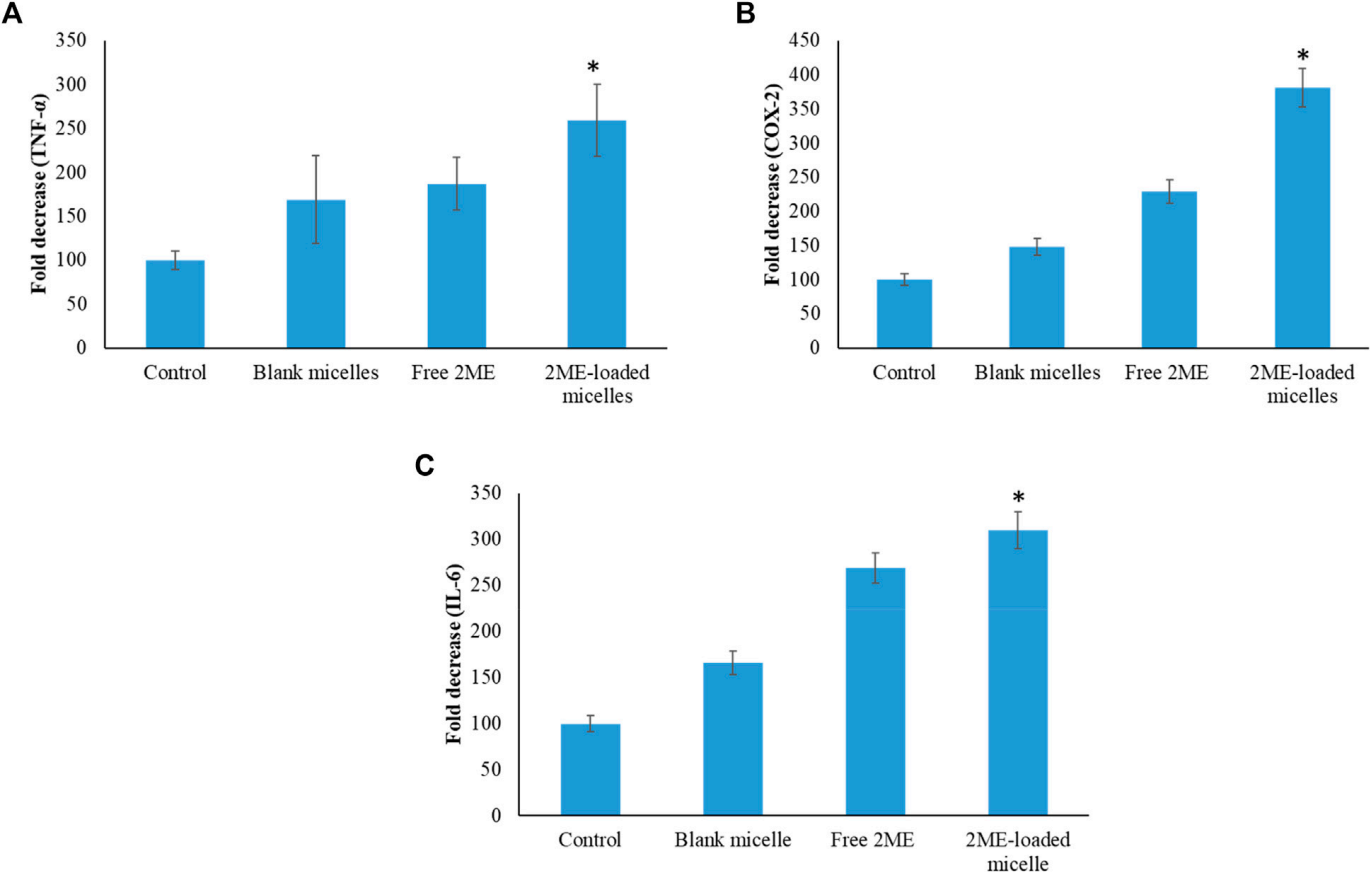
Change in the expression levels of TNF-α **(A)**, COX-2 **(B)**, and IL-6 **(C)** on the PC-3 cells following incubation with blank micelles, free-2 ME and 2 ME-loaded micelles, and vehicle control cells. * represents significant difference between 2 ME-loaded micelles vs other formulations group (*p* < 0.05). Values are expressed as mean ± SD (*n* = 3).

In order to establish the molecular mechanism of the improved anticancer potential of the 2 ME-loaded micellar delivery system, further analysis on molecular markers was performed using the ELISA method. According to the results depicted in Figures 9,10, there is upregulation of caspase-9, p53, and NO whereas the levels of NF-κβ, TNF-α, COX-2, and IL-6 were found to be downregulated. Downregulation of TNF-α and upregulation of tumor suppressor gene, p53, within the cancerous cells were found to be significantly changed when the cells were incubated with the treatments, where the maximum response was obtained from the 2 ME-loaded micelles. This significant alteration of the expressions might aid in the management of prostate cancer conditions (McKenzie and Kyprianou, 2006; Wang and Lin, 2008). Similarly, downregulation of inflammatory markers, such as COX-2, IL-6, and NO, might be important in the management of the cancer condition, as these inflammatory markers are known to play important role in prostate cancer biology. Further, increased permeability of the cells with loss of mitochondrial membrane potential might be correlated to the upregulation of caspase-9, which simultaneously could facilitate the apoptosis of the cancerous cells (McKenzie and Kyprianou, 2006).

Downregulation of NF-κβ within the prostate cancer cells following incubation with treatments might be correlated to the decrease in antiapoptotic effect of NF-κβ, which would further decrease the development of resistance against chemotherapy and radiotherapy (McKenzie and Kyprianou, 2006). These judicious responses of the micellar formulation of 2 ME might be because of the presence of TPGS and 2 ME within the nanocarrier, which synergistically acts toward the improvement of efficacy.

## Conclusion

In summary, the micellar delivery of 2 ME was successfully developed with TPGS and Phospholipon 90G and thereafter optimized using Statgraphics software to obtain a spherical micellar structure of 152 ± 5.2 nm size. Developed micelles showed a sustained and almost complete release of 2 ME from the micellar structure within the time frame of 24 h. Higher entrapment and sustained release profile of 2 ME from the micellar formulation was found to increase apoptotic potential against PC-3 cell line, which on further investigation, was further correlated to an increased level of BAX protein and decreased BCL-2 level. Cell cycle analysis revealed the significant arrest of the PC-3 cells in the G2-M phase following incubation with 2 ME formulation, with a significant increase in cells in the pre-G1 phase. Further, the apoptosis potential of the 2 ME-loaded micelles was correlated to the loss of mitochondrial membrane potential because of generating ROS. Estimating the molecular markers revealed an upregulation of caspase, p53, NO, and downregulation of TNF-α, NF-κβ, COX-2, and IL-6 expressions of the PC-3 cells following incubation with 2 ME micelles formulation when compared with the free drug. Overall, the findings provide a novel platform for developing a nanocarrier of 2 ME for improved therapeutic efficacy of 2 ME for the treatment of prostate cancer.

## Data Availability

The original contributions presented in the study are included in the article/Supplementary Material, further inquiries can be directed to the corresponding author.
