# Supplementary material for: Development, Optimization and Evaluation of 2-Methoxy-Estradiol Loaded Nanocarrier for Prostate Cancer
Source: Front Pharmacol. 2021 Jul 16;12:682337. doi: 10.3389/fphar.2021.682337 (PMC8322574; doi:10.3389/fphar.2021.682337)
Supplement: Supplementary file 1 [file Image1.pdf]

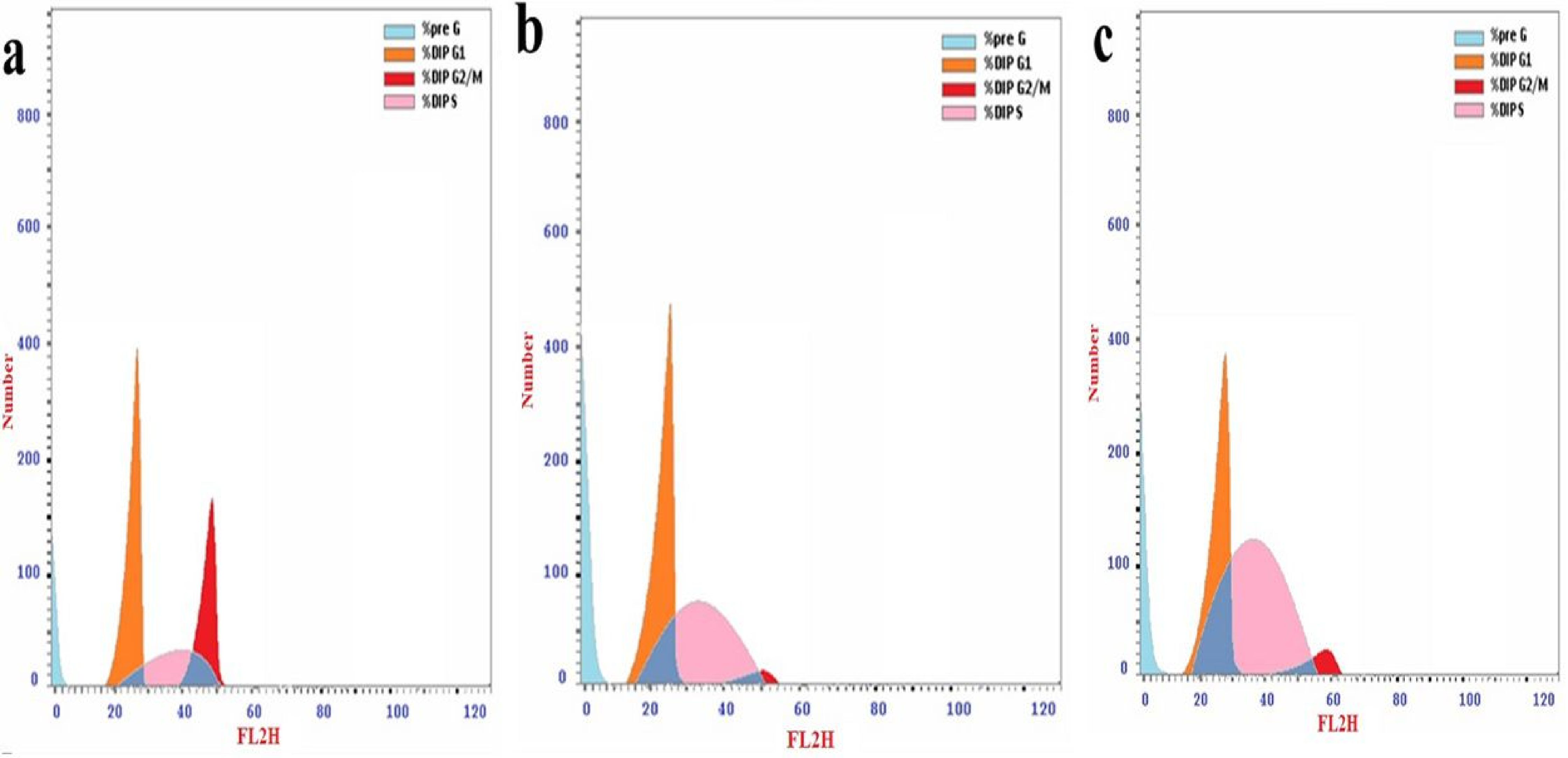

Supplementary figure 1: Representative graph of the cell cycle assay for blank micelles (a), 2ME loaded micelles (b), and free 2ME (C)
